# Perceived Intensity and Discrimination Ability for Lingual Electrotactile Stimulation Depends on Location and Orientation of Electrodes

**DOI:** 10.3389/fnhum.2017.00186

**Published:** 2017-04-21

**Authors:** Joel Moritz Jr., Philip Turk, John D. Williams, Leslie M. Stone-Roy

**Affiliations:** ^1^Department of Mechanical Engineering, Colorado State UniversityFort Collins, CO, USA; ^2^Department of Statistics, Colorado State UniversityFort Collins, CO, USA; ^3^Department of Biomedical Sciences, Colorado State UniversityFort Collins, CO, USA

**Keywords:** sensory substitution, tongue, somatosensation, receptive fields, bioengineering, human, psychophysics

## Abstract

Malfunctioning sensory systems can severely impact quality of life and repair is not always possible. One solution, called sensory substitution, is to use another sensory system to bring lost information to the brain. This approach often involves the use of bioengineered devices that electrically stimulate somatosensory fibers. Interestingly, the tongue is an ideal location for electrotactile stimulation due to its dense innervation, moisture, and protected environment. Success with transmitting visual and vestibular information through the tongue indicates promise for future applications. However, sensitivity and discrimination ability varies between individuals and across the tongue surface complicating efforts to produce reliable and consistent sensations. The goals of the present study were to investigate these differences more precisely to better understand the mechanosensory innervation of the tongue so that future electrotactile devices can be designed more effectively. Specifically, we tested whether stimulation of certain regions of the tongue consistently result in better perception, whether the spacing of stimulating electrodes affects perceived intensity, and whether the orientation of electrodes affects perceived intensity and discrimination. To test these hypotheses, we built a custom tongue stimulation device, recruited 25 participants, and collected perceived intensity and discrimination data. We then subjected the data to thorough statistical analyses. Consistent with previous studies, we found that stimulation of the anterior medial tongue region was perceived as more intense than stimulation of lateral and posterior regions. This region also had the best discrimination ability for electrodes. Dividing the stimulated tongue area into 16 distinct regions allowed us to compare perception ability between anterior and posterior regions, medial and lateral regions, and the left and right sides of the tongue. Stimulation of the most anterior and medial tongue resulted in the highest perceived intensity and the best discrimination ability. Most individuals were able to perceive and discriminate electrotactile stimulation better on one side of the tongue, and orientation of stimulating electrodes affected perception. In conclusion, the present studies reveal new information about the somatosensory innervation of the tongue and will assist the design of future electrotactile tongue stimulation devices that will help provide sensory information to people with damaged sensory systems.

## Introduction

Sensory substitution is the process of using an intact sensory system to gather information that is unavailable via a different sensory system due to damage. The use of Braille by people who are blind is an example of this approach. Currently, multiple groups are investigating the use of specific sensory substitution devices to aid people with sensory deficits. These devices typically include a sensor, to detect specific information, a processor to convert this information into electrical signals, and a stimulator that transmits the information to an intact sensory system (Ward and Wright, 2014). Some of the earliest studies investigating the utility of modern sensory substitution devices involved presentation of visual information to blind individuals via patterns of vibration to the skin of the back (Bach-y-Rita et al., 1969). Later reports showed that visual, proprioceptive and auditory information could be conveyed using sensory substitution devices (Lynch et al., 1988, 1989a,b; Galvin et al., 1991; Wildenberg et al., 2013; Maidenbaum et al., 2014; Novich and Eagleman, 2015). Somatosensory stimulation of the tongue using electrotactile devices is especially promising. The tongue is densely innervated, has good conductivity due to saliva and its tissue properties, and is in a protected environment (Bach-y-Rita, 2004). The dense innervation results in small receptive fields that enable the detection and discrimination of closely spaced mechanical or electrotactile stimuli (Trulsson and Essick, 1997). In fact, multiple groups are working to develop and test tongue stimulation devices to help people with a variety of sensory disorders. These include Kaczmarek (2011) who developed The Tongue Display Unit, multiple groups investigating vestibular substitution and biofeedback, and Danilov and Tyler (2005) who developed the BrainPort® (Tyler et al., 2003; Vuillerme and Boisgontier, 2009; Barros et al., 2010; Wildenberg et al., 2013). These devices have been used in multiple studies including vision and balance substitution, neuroplasticity applications and augmentation of sensory information.

Despite the increasing use of electrotactile stimulation of the tongue for sensory substitution applications, there is little detailed information about the regional variability of the tongue to this type of stimulation and few studies have investigated how to increase effectiveness of stimulation using alternative electrode array designs. Previous studies have been small (averaging eight participants each), and mostly centered on mapping the sensitivity of the tongue without analyzing electrotactile two-point discrimination ability (Lozano et al., 2009; Tyler et al., 2009; Wilson et al., 2012). These studies indicate that anterior-medial tongue regions have lower thresholds for electrotactile stimulation relative to posterior regions and sensitivity to electrotactile stimulation varies widely between individuals. How regional and individual variabilities for perceived electrotactile stimulation relate to tongue physiology is just beginning to be investigated. For example, individuals are better able to discriminate electrotactile stimuli at higher intensities, which may be due to recruitment of additional somatosensory fibers with stronger stimuli (Lozano et al., 2009).

The goals of the present study are to increase the efficacy of future electrotactile tongue stimulation applications by providing detailed information about electrotactile sensitivity and discrimination abilities across the tongue surface in a large participant pool, and uncover specific patterns that can be used for future electrotactile electrode array design by subjecting the data to thorough statistical analysis. Specifically, we first confirmed that our custom tongue stimulation device, experimental protocol and analysis approach produced results consistent with previously published studies. Next, we designed experiments to determine whether the positions of active electrodes affect perceived intensity and discrimination of stimulation. Specifically, we tested: (1) Whether specific 1 cm^2^ regions of the tongue are more sensitive to electrotactile stimulation relative to other regions; (2) Whether specific 1 cm^2^ regions of the tongue are better able to discriminate 2 active electrodes presented at a constant voltage relative to other regions; (3) Whether closely spaced electrodes are perceived as more intense than those that are spaced further apart; and (4) Whether the orientation of two active electrodes affects perceived intensity or discrimination ability.

## Materials and Methods

### Subjects

Twenty-five healthy adults, 14 males and 11 females, volunteered to participate in these experiments. The subjects were recruited with fliers posted around Colorado State University campus and most were students or employees of the university. Participants filled out a questionnaire prior to being admitted into the study. This helped avoid including subjects that had mouth sores, infection, or external devices that might interfere with electrotactile stimulation or perception. Additionally, it was requested that subjects be non-smokers to decrease the possibility of pre-existing oral tissue damage which might affect results. Once admitted into the study, researchers led each subject through an informed consent process, which included a consent form which was initialed and signed by both the participant and researcher in accordance with the Declaration of Helsinki. The subject was then assigned a code that was used to identify data corresponding to that individual. Subjects were compensated for their participation and paid $8.50/h. One trial typically took an hour and 1–4 trials were done per participant. Only data from the first trial of each participant were included in the current manuscript. All procedures and forms were approved by the Institutional Review Board at Colorado State University.

### Tongue Stimulation Devices

**Figure 1:**
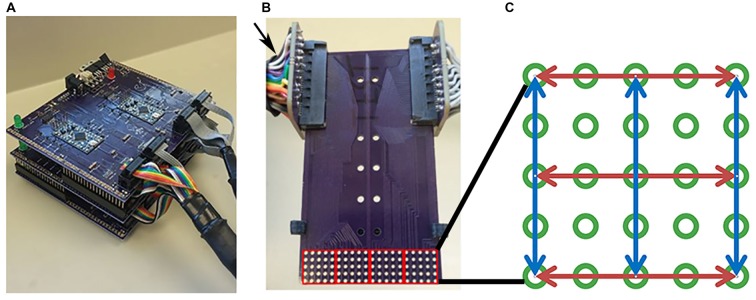
**Tongue stimulation device and mouthpiece. (A)** Picture of Cthulhu device. **(B)** Sample mouthpiece used in experiments. Arrow indicates the card slot connectors at the end of cables attached to the outputs of the Tickler or Cthulhu device. The electrode array consisted of 5 × 20 electrodes that were divided into four 5 × 5 subarrays (red squares) during analyses. **(C)** Diagram of an individual subarray. Red and blue arrows indicate the 3 rows and 3 columns of electrodes chosen for testing. Active electrodes in each row or column were 2, 4, 6, or 8 mm apart.

Two tongue stimulation devices were designed and constructed prior to experiments as described by Moritz (2017). Briefly, both devices were based on published designs (Kaczmarek, 2011) and utilized positive stimulation pulses and an RC network to minimize net DC current through tissue by adding a small negative voltage pulse following each positive pulse. One device, “The Tickler”, was constructed first for preliminary investigations, and a second device, “The Cthulhu”, was based on the initial device, but included improvements which made it sturdier for increased use and handling by multiple researchers (Figure 1A). Both devices were maintained at a 5-volt Pulse Amplitude (PA) for all experiments. Devices were assembled by Sapien, LLC (Fort Collins, CO, USA) using supplies purchased from Mouser Electronics (Mansfield, TX, USA).

### Mouthpiece Arrays

Electrode arrays were made from simple 2-layer FR-4 printed circuit boards designed in eagle PCB design software and produced by OSH Park, LLC (Lake Oswego, OR, USA) as described previously (Moritz, 2017). Electrodes were gold plated vias with an outer diameter of 1.016 mm and an annular ring thickness of 0.06 mm. The electrodes were positioned in a 5 × 20 rectangular array with 2 mm center-to-center spacing (approximately 1 mm edge-to-edge). The completed rectangular array measured 1 cm by 4 cm and was located at one end of the circuit board (Figure 1B). A stop pad was designed to be clipped into the circuit board such that the electrode array could be positioned precisely at one of four locations on the tongue surface. This allowed us to stimulate, analyze and map a 4 cm^2^ region of the anterior tongue. Arrays and stop pads were designed to be cheap and disposable so that each participant could be assigned a mouthpiece array for his/her personal use throughout the study. This minimized the possibility of disease transmission. Mouthpieces and stop pads were sterilized prior to use. Mouthpieces were washed with detergent, rinsed in distilled water, immersed in non-chlorine bleach for 100 s, rinsed with distilled water and boiled for 100 s. Stop pads were manufactured on an FDM style 3D-printer and extruded at 230°C onto a heated platform maintained at 110°C. Sterilized tongs were used to move both mouthpieces and stop pads into individual sterile petri dishes. These were wrapped in Parafilm and stored at room temperature until used in experiments.

### Waveform Characteristics of Electrotactile Stimulation

The waveform used to stimulate the tongue in this study were based on results of previous studies indicating that multiple biphasic pulses grouped in short bursts result in comfortable, effective electrotactile stimulation (Kaczmarek and Tyler, 2000; Tyler et al., 2009; Kaczmarek, 2011). For our studies, we used a constant value of 5 volts for PA and an Outer Burst Period (OBP) of 36 ms, a PA of 5 volts, an Inner Burst Number (IBN) of 3, a Peak to Peak (PP) length of 10 μs, and an Inner Burst Period (IBP) of 150 μs. Pulse Width (PW), and Outer Burst Number (OBN) correlate with effective perception and comfort of the stimulus (Moritz, 2017) and we found that an effective stimulation setting for different participants could reliably be determined by coupling these two parameters and simultaneously incrementing their values from 3 to 9 (unpublished observations). This resulted in seven distinct noticeability settings which could be used to select the most effective stimulation for each subject. Thus, one of these settings was selected by each participant for the duration of the study so that we could assess perception of intensity and discrimination at a constant setting. During experiments, only one electrode was active at a time to improve the localization of sensations and provide a return path via inactive electrodes (Kaczmarek, 2011; Wilson et al., 2012). For 2-point discrimination tests, the time between the activation of one electrode and the activation of the other was less than 35 ms and not detectable by the participant.

### Electrotactile Testing

For each experiment, the subject was aided by an investigator to insure proper placement of the mouthpiece and recording of data. Subjects placed the mouthpiece consisting of an electrode array and stop pad in their mouths with the stop pad indexed to the first position so that the anterior tip of the tongue was stimulated first. At the end opposite the electrode array, the circuit board was plugged into two card slot connectors at the end of cables attached to the outputs of the Tickler or Cthulu device (Figure 1B).

Prior to each set of experiments, individual subjects worked with the investigator to determine a comfortable, but strong effective perception setting for electrotactile stimulation as described earlier. This setting was maintained for the duration of the experiment so that any changes in perceived intensity or discrimination ability by the participant would reflect differences in somatosensory ability. Once the preferred setting was determined, the investigator began the electrotactile analysis. Stimuli were presented to the participant after he/she indicated readiness by a voice or hand signal. The researcher then pressed one button on the computer, which resulted in the presentation of one or two active electrodes in a specific region of the mouthpiece. The participant was asked to record whether zero, one, two, or more, discrete sensations were felt. Thus, if a subject reported 0 discreet sensations, this would indicate that the active electrode was not perceived and therefore unable to stimulate somatosensory fibers in that specific region at the specified setting chosen for the participant. A response of more than two stimulations, or two stimulations when only one was presented, suggested that the somatosensory system was spontaneously active, other stimuli were present and unaccounted for, or possible malingering by the subject. Participants were instructed to record responses within 2 s of onset since previous studies indicated that electrotactile sensations fade rapidly. In addition to perceived number of electrotactile stimuli, each subject rated the perceived intensity of the sensation(s) on a scale from 0 to 10, 0 being no perceived sensation, and 10 being a very intense sensation. This produced data about the sensitivity of specific tongue regions since a higher perceived intensity at a constant electrode setting for identical stimuli at different tongue locations suggests more sensitivity to stimulation in regions with higher perceived intensity. The investigator then proceeded to the next preset stimulus and the process was repeated.

Stimuli presented to the subject were pre-programmed in the stimulation devices in the form of lists stored on the device firmware. These lists were generated by first dividing the full 5 × 20 array into four 5 × 5 sub-arrays as shown in Figure 1B (red squares). Then, 3 rows and 3 columns of electrodes were selected in each sub-array as shown by the blue and red lines in Figure 1C. In each row and each column, four pairs of electrodes that were 8, 6, 4, or 2 mm center to center apart were recorded in a Microsoft Excel spreadsheet. The electrode pairs were centered in their respective row or column, but due to geometric constraints, may have been shifted off-center in the row or column by 2 mm. The order of these row/column pairs were randomized in Excel and mixed with another list of 20 random, single electrodes. This gave 116 specific one or two electrode stimuli that were tested. One list of the 116 stimulus patterns was generated and randomized for each of the four positions at which the array was positioned in the mouth. The same four randomized lists were used for each participant and these lists were stored in device firmware. List 1 consisted of the electrodes that were activated when the mouthpiece was placed at the first position at the anterior tip of the tongue up to list 4, which was the list of electrodes that were activated when the mouthpiece was indexed to the most posterior region tested. This gave 464 separate patterns across a 4 cm by 4 cm area of each subject’s tongue.

### Data Analysis and Statistics

Participant responses to stimuli were manually entered into a custom designed Excel spreadsheet, which cross-referenced these responses against the list of activated electrode(s), their position on the array, and the position of the array on the tongue. To represent two-point discrimination data visually, color-coded maps were generated to represent the minimum center to center spacing for which a subject could correctly identify two distinct sensations. Data was taken from the Excel spreadsheet into MatLab for further analyses. For visual representations, perceived intensity and discrimination ability for the two electrotactile points was interpolated between tested loci across the 4 cm^2^ area of the tongue using MatLab software. Each interpolation calculation was iterated until values converged. For visual representations of minimum discrimination ability, discrimination ability was considered to be 10 mm at tested points where participants could not correctly distinguish electrodes which were spaced 8 mm apart. This assumption was only used to make graphical representations and was not used for any statistical analysis.

**Figure 2:**
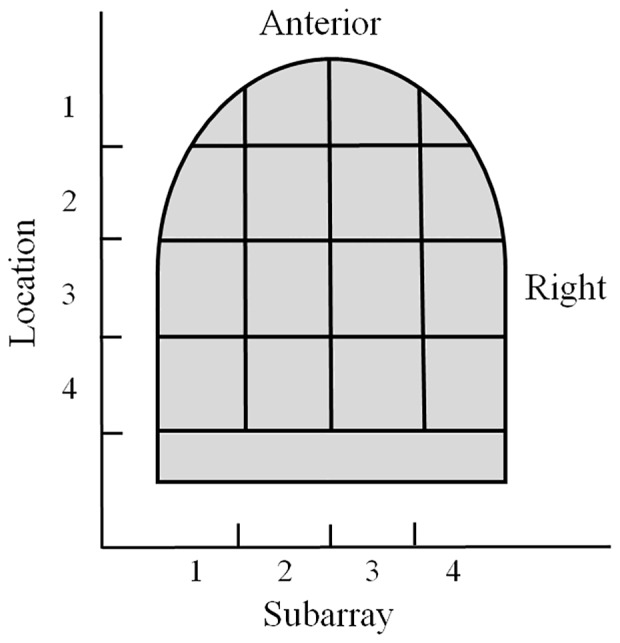
**Illustration of the regional areas used for analyses**. The tongue region stimulated by the mouthpiece of the electrotactile device was divided into 16 areas for analyses. Anterior to posterior sections were referred to as Location 1–4 with Location 1 as the most anterior region. Lateral regions were referred to as Subarrays. There were four Subarrays: 1–4, with Subarray 1 representing the left lateral section of the tongue.

For regional analyses, the electrode array on the mouthpiece was divided into Left-to-Right Subarrays, resulting in four identifiable regions (Figure 1B). In addition, the mouthpiece was positioned in four anterior-to-posterior Locations on the tongue. Using Subarray and Location information, there were 16 identifiable regions of the tongue that were exposed to electrotactile stimulation in our study (Figure 2). For statistical analyses, factors and their levels were defined as Location (1, 2, 3, 4, from the anterior/front to the posterior/back of the tongue, respectively), Subarray (1, 2, 3, 4, from the left-hand side to the right-hand side of the tongue, respectively), Distance (2, 4, 6, 8 mm) between electrodes within a pair, and Orientation (vertical or horizontal) of electrode pairs.

For both the perceived level of intensity of stimulation and the perceived number of distinct points of stimulation, subjects reported observations on a rating scale which corresponded to {0, 1, 2, …, 10} and {0, 1, 2, 3}, respectively. It should be noted that during the test, only one or two electrodes were activated each time a participant response was elicited and a subject response of 0 or 3 electrodes indicated the presence of perceptual or physiological phenomena. We computed the means of the three row/column subsample observations, holding the levels of Subject, Location, Subarray, Distance and Orientation fixed, and used these as our response variables for the data analysis. There were 25 subjects yielding a total of 3200 observations. Exploratory data analysis was performed generating summary statistics and graphics. A linear mixed model was fit to both responses where the factors above were fixed effects and random effects were defined corresponding to experimental units in the study (e.g., Subjects). Several interactions of interest were also included in the model. The corrected Akaike Information Criterion (AICc; Burnham and Anderson, 2002) was used to model the covariance structure of the errors among levels of Distance. Standard residual diagnostic plots were used to check the assumptions of the model. All data analysis was done using SAS for Windows software, Version 9.4. A significance level of 0.05 was used throughout the data analysis for hypothesis testing.

## Results

### Main Effects and Interaction between Variables for Perceived Intensity

Perceived intensity was significantly higher in the anterior 2 cm of the tongue (*P* < 0.0001; Table 1). We first investigated the subject-to-subject variation in this anterior region. To do this, the linear mixed model included a random effect for each subject and the subject-to-subject variance component was estimated to be 0.8927 with a Wald-based 95% confidence interval of (0.4978, 2.0458), indicating that perceived intensity for electrotactile stimuli varied substantially from person-to-person. To address smaller/greater Subarray variation depending upon the individual tested, we included a second random effect in the model associated with Subarray (1 vs. 2 vs. 3 vs. 4) for each Subject. The variance component for these random Subarray effects was estimated to be 0.3786 with a Wald-based 95% confidence interval of (0.2637, 0.5897), indicating that perceived intensity varied between Subarray regions and this depends upon the Subject. Thus, stochastic variation depended upon the Subject and Subarray differences depended upon the Subject.

**Table 1:** **The estimated mean perceived intensity for each Location-Subarray region**.

Location	Subarray	Estimate
1	1	1.1304
1	2	3.3637
1	3	3.7679
1	4	1.8087
2	1	1.1787
2	2	1.9137
2	3	1.9604
2	4	1.4021
3	1	0.7587
3	2	1.0854
3	3	1.0304
3	4	0.7771
4	1	0.6937
4	2	0.7154
4	3	0.8104
4	4	0.6287

*Standard error of the estimate was 0.1986 in all cases. The estimated mean perceived intensity for Locations 1 and 2 was 2.0657 and 0.8125 for Locations 3 and 4*.

### Main Effects and Interaction between Variables for Discrimination Ability

Overall, with respect to discrimination ability, participants were unable to distinguish two electrotactile stimuli on the posterior two centimeters of the tested region in contrast to the anterior half of the tested region (*P* < 0.0001; Table 2). For the anterior two cm of the tongue, the variance component for the random Subject effects was estimated to be 0.0493 with a Wald-based 95% confidence interval of (0.0252, 0.1372), indicating that in our 25 subject pool, the perceived number of electrodes varied substantially between subjects. To address smaller/greater Subarray variation depending on the Subject, we included a second random effect associated with Subarray (1 vs. 2 vs. 3 vs. 4) for each Subject. The variance component for these random Subarray effects was estimated to be 0.0383 with a Wald-based 95% confidence interval of (0.0267, 0.0595). Thus, stochastic variation depended upon the Subject and Subarray differences depended upon the Subject.

**Table 2:** **The estimated mean perceived number of electrotactile stimuli for each Location and Subarray region**.

Location	Subarray	Estimate
1	1	0.6157
1	2	1.1073
1	3	1.2740
1	4	0.7207
2	1	0.6390
2	2	0.8557
2	3	0.9090
2	4	0.7323
3	1	0.5257
3	2	0.6840
3	3	0.6890
3	4	0.5223
4	1	0.4973
4	2	0.4957
4	3	0.5757
4	4	0.4773

*Standard error of the estimate was 0.0763 in all cases. The estimated mean perceived number of electrotactile stimuli for Locations 1 and 2 was 0.8567 units and 0.2983 for Locations 3 and 4*.

### Sensitivity to Electrical Stimulation was Consistent with Previous Studies

**Figure 3:**
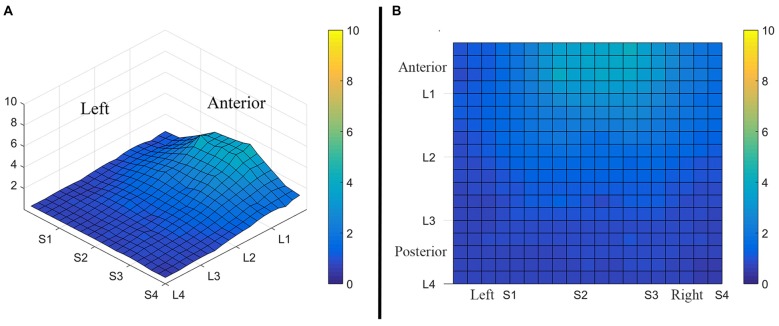
**Average perceived intensity for all subjects at all discrimination distances across the tongue region. (A)** Three dimensional map of the tongue. **(B)** Two dimensional map of the same data shown in **(A)**. The average perceived intensity for all subjects was highest in the most anterior region (stimulated by electrodes in Location 1) and decreased for the more posterior regions of the tested area (Locations 3 and 4). The average perceived intensity appeared to be greatest just lateral to the midline area in tongue regions stimulated by electrodes in Subarrays 2 and 3. Scale indicates intensity ratings recorded by subjects in response to electrotactile stimulation with 0 indicating no perceived stimulation (dark blue) and 10 indicating the maximum averaged perceived intensity for this group (yellow).

**Figure 4:**
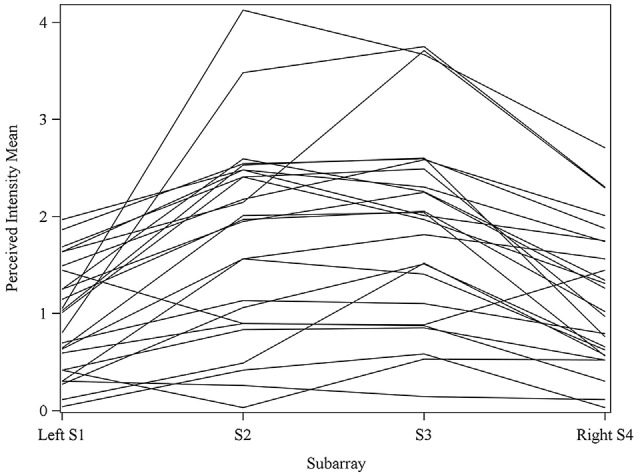
**Perceived intensity profiles for all 25 subjects relative to Subarray across the tongue**. Mean perceived intensities at each Subarray are indicated for each subject. Note that the profiles have a tendency to have large perceived intensity values in the middle of the tongue, regions stimulated by electrodes in Subarrays 2 and 3. In contrast, more lateral regions, stimulated by electrodes in Subarrays 1 and 4, have lower perceived intensities.

**Figure 5:**
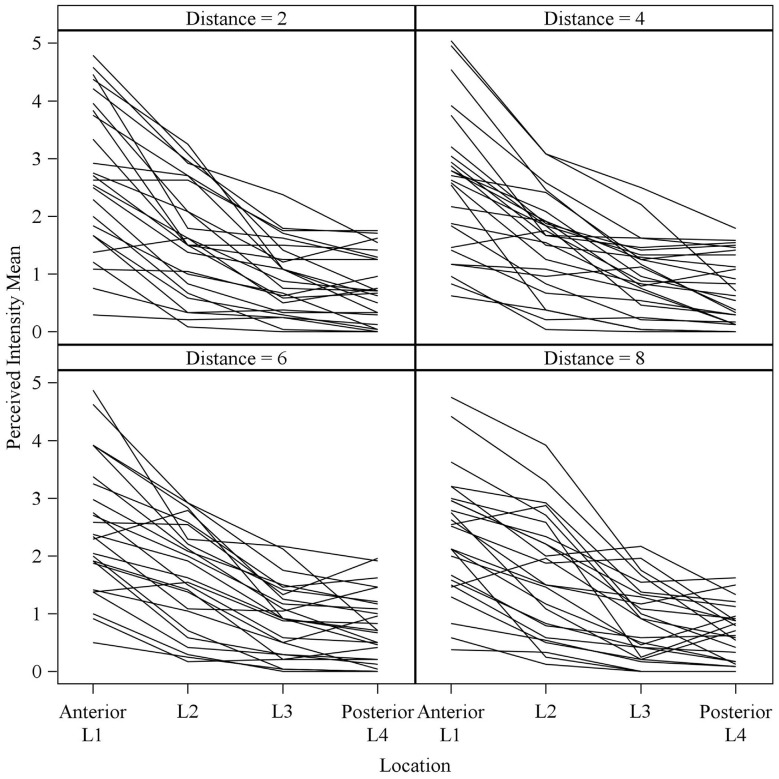
**Mean perceived intensities for all 25 subjects at different anterior to posterior locations on the tongue for each different electrode spacing (2, 4, 6 and 8 mm center to center distance)**. Each line reflects an individual participant’s mean perceived intensity at each of four locations. Location 1 is the most anterior tongue region tested and location 4 is the most posterior tongue region tested. Note that for each electrode spacing distance, the profiles generally trend downward from the anterior tip of the tongue (left side of each figure, Location 1) to the most posterior region tested (right side of each figure, Location 4).

**Figure 6:**
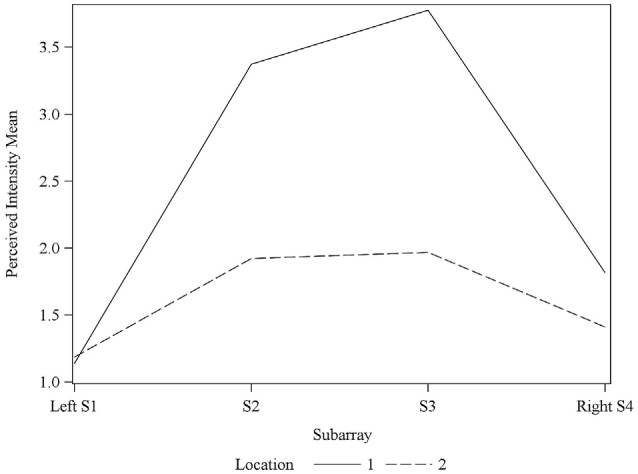
**Average perceived intensity for Locations 1 and 2 relative to Subarray**. At Location 1, the average perceived intensity was lower for the left side of the tongue, relative to the right side. The left side of the tongue is the area stimulated by electrodes in Subarrays 1 and 2 and the right side is stimulated by electrodes in Subarrays 3 and 4. For Location 2, there was no statistical difference between perceived intensity for the left vs. the right side.

Consistent with findings from studies using a variable voltage stimulus (Tyler et al., 2009), our analysis revealed that Location and Subarray both affect perceived intensity (*P* < 0.001) for a constant voltage stimulus across the tongue. On average, the perceived intensity of a constant voltage stimulus on a participant’s tongue was reported as highest in the anterior most medial region and was perceived as less intense in more posterior and lateral regions (Table 1, Figures 3–5). An increase in perceived intensity in Location 1 compared to Location 2 was found to be highly significant in the medial regions of the tongue, but not in the lateral regions of these Locations (*P* < 0.001; Figure 6, Table 3).

**Table 3:** ***F*-tests for differences between Locations 1 and 2 at each of the four Subarrays with respect to Perceived Intensity**.

	Tests of effect slices				
Effect	Subarray	Num DF	Den DF	*F* value	Pr > *F*
Location * Subarray	1	1	81	0.11	0.7370
Location * Subarray	2	1	81	102.22	<0.0001
Location * Subarray	3	1	81	158.84	<0.0001
Location * Subarray	4	1	81	8.04	0.0058

*Examination of the differences between Locations 1 and 2 at each of the four Subarrays revealed huge differences between Location 1 and 2 for Subarrays 2 and 3*.

### Two Point Discrimination was Consistent with Previous Studies

**Figure 7:**
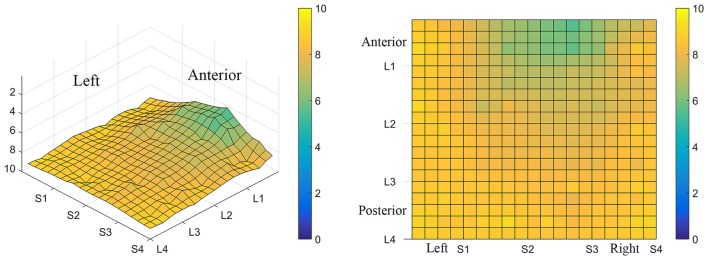
**Average minimum discrimination distances for all participants**. The ability to discriminate lingual electrotactile stimuli at 2, 4, 6 and 8 mm apart was determined for each participant. These data were then averaged to produce the maps shown here. Regions with the highest acuity tested (participants were able to discriminate stimuli that were 2 mm apart) are represented by green and regions with low acuity (participants were unable to discriminate stimuli at 8 mm apart, the largest distance tested) are represented by light yellow areas as indicated by the color scale to the right of each map. On average, the anterior-medial region (between S1 and S3 on these maps) shows the highest acuity and thus has the lowest two point discrimination threshold.

**Figure 8:**
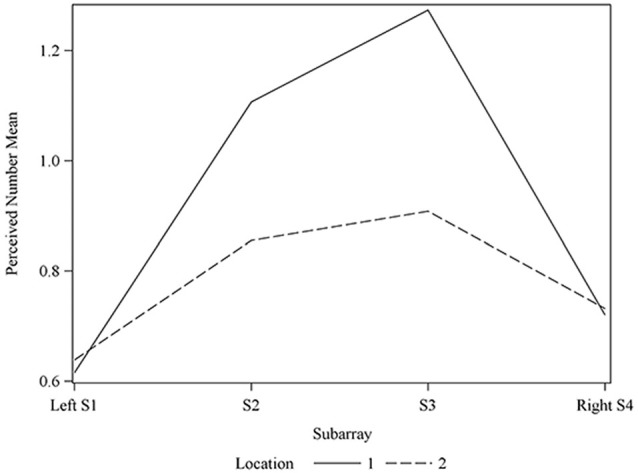
**Average perceived electrode number**. For Locations 1 and 2 relative to Subarray. At Location 1, the average perceived number of electrodes was lower for the left side of the tongue, relative to the right side. The left side of the tongue is the area stimulated by electrodes in Subarrays 1 and 2 and the right side is stimulated by electrodes in Subarrays 3 and 4. For Location 2, there was no statistical difference between perceived electrode number for the left vs. the right side.

Consistent with previous studies (Maeyama and Plattig, 1989), our results show that perceived number of stimuli in a two point discrimination test is affected by the location of the stimuli along the anterior-posterior axis of the tongue (Location; *P* = 0.0011) and the location of stimuli along the lateral-to-lateral axis of the tongue (Subarray; *P* < 0.0001; Table 4). On average, the lingual discrimination ability for participants was best in the anterior medial region and discrimination decreased in more posterior and lateral regions (Table 2, Figure 7). An increase in perceived number of stimuli in Location 1 compared to Location 2 was found to be highly significant in the medial regions of the tongue, but not in the lateral regions of these Locations (*P* < 0.0001; Figure 8, Table 5). These findings are consistent with previous reports for both mechanical and electrotactile stimulation (Maeyama and Plattig, 1989; Trulsson and Essick, 1997).

**Table 4:** ***F*-tests of fixed effects in model for perceived number of electrotactile stimuli in the anterior 2 cm of the tongue**.

	Tests of fixed effects			
Effect	Num DF	Den DF	*F* value	Pr > *F*
Location	1	24	13.82	0.0011
Subarray	3	72	25.21	<0.0001
Distance	3	1191	1.81	0.1431
Orient	1	271	6.83	0.0095
Location * Subarray	3	271	28.91	<0.0001
Location * Distance	3	1191	0.22	0.8818

*Location, Subarray, their interaction and Orientation are important with respect to their effects on the mean*.

**Table 5:** ***F*-tests for differences between Locations 1 and 2 at each of the four Subarrays with respect to perceived number of electrotactile stimuli**.

	Tests of effect slices				
Effect	Subarray	Num DF	Den DF	*F* value	Pr > *F*
Location * Subarray	1	1	62.1	0.22	0.6427
Location * Subarray	2	1	62.1	25.29	<0.0001
Location * Subarray	3	1	62.1	53.19	<0.0001
Location * Subarray	4	1	62.1	0.05	0.8164

*Note that discrimination ability was different between Locations 1 and 2 for Subarrays 2 and 3*.

### Effect of Electrode Spacing on Perceived Intensity

To test our hypothesis that the spacing of two active electrodes influences the perceived intensity of stimulation, we analyzed this effect statistically. Based on our analysis, the distance between two active electrodes affects perceived intensity and depends on the Location of the stimulus on the tongue (*P* = 0.0155). There was large variation in sensitivity across the surface of the tongue (Figure 5) with very low sensitivity in the posterior half of the tested area (Figure 3). We suspected that the effect of electrode spacing on perceived intensity might be different for the area of the tongue with higher sensitivity and therefore confined a portion of the analysis to Location 1. Analysis using differences among least squares means allowed us to conduct multiple comparisons between the different electrode distances with respect to mean perceived intensity in Location 1 (Table 6). There was a statistically significant difference in perceived intensity for stimuli that were 2 mm apart relative to those that were 6 or 8 mm apart (*P* = 0.0170 and 0.0006, respectively), and for stimuli that were 4 mm apart relative to stimuli that were 8 mm apart (*P* = 0.0432; Table 6). For Location 1, the most anterior region tested, the distance between electrodes had a significant effect on perceived intensity (*P* < 0.0001; Table 7). In general, reported values for perceived intensity of stimuli decreased as the space between electrodes increased from 2 mm to 8 mm (Table 8).

**Table 6:** **Differences among estimated perceived intensity means were used to conduct multiple comparisons between distances at Location 1**.

Distances	Estimate	*t* value	Pr > |t|
2 vs. 4	0.1562	2.12	0.1017
2 vs. 6	0.2155	2.93	0.0170
2 vs. 8	0.3439	4.67	0.0006
4 vs. 6	0.0592	0.80	0.4209
4 vs. 8	0.1877	2.55	0.0432
6 vs. 8	0.1285	1.75	0.1620

*Distances are in mm and indicate the center-to-center electrode spacing. Estimated perceived intensity means were 2.6966, 2.5404, 2.4811, and 2.3527 for Distances 2, 4, 6, and 8, respectively. Per Holm adjusted P-values, electrodes that were 2 mm apart were perceived as more intense than electrodes that were 6 or 8 mm apart and electrodes that were 4 mm apart were perceived as more intense than electrodes that were 8 mm apart. Standard error of the estimate was 0.0736 in all cases*.

**Table 7:** ***F*-tests for differences among Distances with respect to mean perceived intensity at each Location**.

	Tests of distance effect at each location			
Location	Num DF	Den DF	*F* value	Pr > *F*
1	3	2385	7.52	<0.0001
2	3	2385	0.82	0.4851
3	3	2385	1.12	0.3396
4	3	2385	1.30	0.2744

*Note that the only statistically significant result is for Location 1*.

**Table 8:** **Estimated mean perceived intensity for electrodes spaced at 2, 4, 6 or 8 mm apart in Location 1**.

Location	Distance	Estimate
1	2	2.6966
1	4	2.5404
1	6	2.4811
1	8	2.3527

*As the space between electrodes increased from 2 mm to 8 mm, there was a small, but statistically significant decrease in the perceived intensity of the stimulation. Standard error of the estimate was 0.1800 in all cases*.

### Effect of Electrode Orientation on Perceived Intensity

We suspected that the orientation of two active electrodes may have an effect on perceived intensity, regardless of whether or not the two electrodes were correctly discriminated. We analyzed perceived intensity relative to the orientation of electrodes in the entire 4 cm^2^ tested area and in the most anterior 2 cm of the tongue (Table 9) and discovered that orientation of electrodes did not affect perceived intensity (*P* = 0.8243 and 0.5866, respectively).

**Table 9:** ***F*-tests of fixed effects in model for perceived intensity in the anterior 2 cm of the tongue**.

	Tests of fixed effects			
Effect	Num DF	Den DF	*F* value	Pr > *F*
Location	1	24	75.55	<0.0001
Subarray	3	72	36.38	<0.0001
Distance	3	1191	2.28	0.0779
Orientation	1	271	0.30	0.5866
Location * Subarray	3	271	58.16	<0.0001
Location * Distance	3	1191	3.73	0.0110

Note the large F values for Location and Subarray indicating that these parameters are important with respect to their effects on mean perceived intensity. These fixed effects also have an interaction as indicated by the significant Location * Distance F value. The distance between electrodes has a mild effect on the mean perceived intensity. In contrast, the orientation of electrodes does not affect perceived intensity.

### Effect of Electrode Orientation on Discrimination Ability

Similarly, we suspected that the orientation of two active electrodes on the surface of the tongue may affect the ability to correctly discriminate that stimulus. As most participants were unable to distinguish two electrotactile stimuli on the posterior two centimeters of the tested region, we confined our statistical analysis of discrimination ability to the anterior half of the tested region. In our two-point discrimination tests, we analyzed the reported number of perceived stimuli relative to the orientation of the two active electrodes. Our results indicate that the orientation of electrode pairs during stimulation had a significant effect on perceived number in the front half of the tongue (*P*-value = 0.0095; Table 4).

We compared electrodes that were oriented parallel to the longitudinal axis of the tongue (anterior to posterior = vertical) to electrodes that were oriented perpendicular to the longitudinal axis of the tongue (lateral to lateral = horizontal). The estimated mean for the horizontal orientation was 0.8796 (95% confidence interval (0.7679 0.9913)) and was 0.8325 (95% confidence interval (0.7208 0.9442)) for the vertical orientation, indicating that discrimination between closely spaced electrotactile stimuli is better if the electrodes are arranged perpendicular to the longitudinal axis of the tongue (horizontal).

### Perceived Intensity Sidedness was Observed in Some Participants

**Figure 9:**
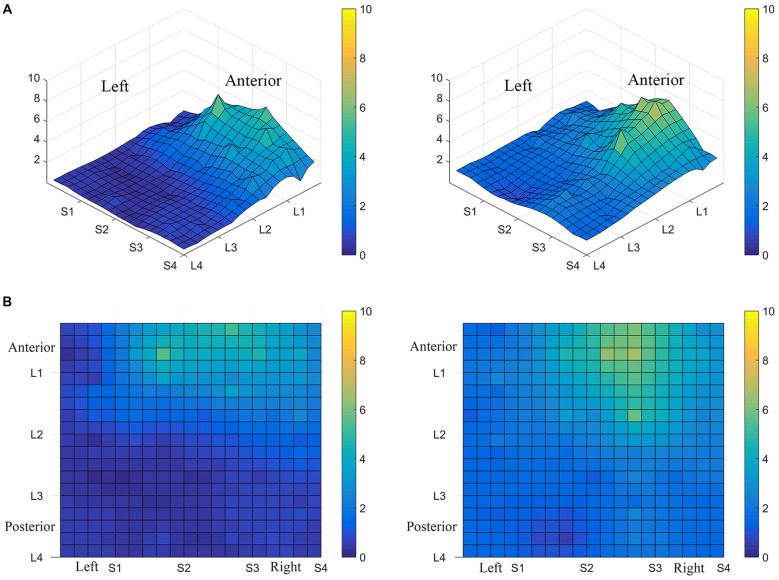
**Perceived electrotactile intensity is often higher on one side of the tongue**. Regional differences in perceived electrotactile intensity are shown for two different subjects **(A,B)**. Top images are 3D maps and bottom images are 2D maps. For both of these participants, electrotactile stimuli were usually perceived as more intense when presented to the right side of the tongue (lighter colors).

Interestingly, the data suggested that there might be left-right asymmetry with respect to perceived intensity for electrotactile stimulation (Figures 3, 6). To investigate this more closely, we analyzed individual intensity maps from all 25 subjects. This analysis revealed that most of the subjects reported more intense sensations on one side of the tongue relative to the other (20/25 participants). For some subjects this perceptual asymmetry was moderate, but for other participants the asymmetry was extreme (Figure 9). We compared the left side of the front half of the stimulated area (regions stimulated by electrodes in Locations 1 and 2, Subarrays 1 and 2) relative to the right side (regions stimulated by electrodes in Locations 1 and 2, Subarrays 3 and 4) via a contrast. On average, perceived intensity was estimated to be 0.3381 units less for the left side of the tongue relative to the right side (*P*-value = 0.0150). Next, we compared the left side to the right side at each of the two anterior Locations (1 and 2). There was no statistically significant difference between the right and left side for Location 2 of the tongue (*P-value* = 0.3611). However, the perceived intensity was estimated to be 0.5413 units less on the left side vs. the right side for tongue regions stimulated by electrodes in Location 1 (*P-value* = 0.0004).

### Discrimination Ability Sidedness was Observed in Some Participants

Consistent with our results of perceived intensity for electrotactile stimulation, the ability to detect closely spaced electrodes was better for one side of the tongue relative to the other side in our participant pool. This was especially evident in anterior regions (Figures 7, 8). To analyze this carefully, we compared the left side of the front half of the stimulated tongue region vs. the right side via a contrast. The mean perceived number was estimated to be 0.1046 units less for the left side relative to the right (*P*-value = 0.0177). Comparison at each of the two Locations revealed that for Location 1, the mean perceived number on the left was estimated to be 0.1358 units less than the right side (*P*-value = 0.0045), whereas at Location 2, no statistically significant difference was noted (*P*-value = 0.1194; Figure 8). Thus, for our participant population, electrode discrimination in the most anterior 1 cm of the tongue was on average, better for the right side of the tongue.

## Discussion

The results from these experiments suggest that perceived intensity for electrotactile stimulation and discrimination is affected by multiple factors including the individual subject, the specific region of the tongue that is stimulated, the spacing between electrodes, and the orientation of electrodes. Stimulation of the most anterior 2 cm of the tongue resulted in the highest perceived intensity and the best discrimination ability under the condition of constant voltage. Furthermore, electrotactile stimulation of medial tongue regions was perceived as more intense than stimulation of lateral regions consistent with previous studies (Maeyama and Plattig, 1989; Tyler et al., 2009; Wilson et al., 2012), Interestingly, it appeared that perception for electrotactile stimuli was best for regions just lateral to the midline rather than in the most medial region and the sensitivity and enhanced discrimination ability of this paramedial region extended beyond the most anterior tip of the tongue (Figures 3, 7). In future experiments, we would like to investigate this trend more closely by further subdividing the 1 cm^2^ tongue regions analyzed in these studies.

Electrotactile perception was best for the most anterior centimeter of the tongue. In this region, electrode distance significantly affected perceived intensity and the distance between correctly discriminated electrodes was smallest. However, the perceptual ability of the lateral anterior tongue regions might have been underestimated since it is possible that the rectangular design of the array could have resulted in electrodes that did not stimulate any tongue region due to interference by the mandibular incisors. This would not have been an issue for medial tongue regions stimulated by electrodes in Subarrays 2 and 3 at Location 1. The use of electrical sensing techniques to verify whether certain electrodes are in contact with the tongue in future studies would be beneficial.

Our data indicate that perceived intensity for electrotactile stimulation and discrimination ability is usually higher for one side of the tongue. Mean data from our 25-subject pool suggests that the right side of the tongue is better able to detect and discriminate electrotactile stimuli. Enhanced electrotactile perception on the right side was not found in all participants however. Analysis of individual tongue maps revealed that although most of the tested participants (20/25) reported more intense sensations and better discrimination for one side of the tongue relative to the other, the right side was not always superior (data not shown). Higher perceived intensity and better discrimination was reported on the right side of the tongue for 16/20 of the participants, on the left side for 4/20 of these participants, and 5/25 reported approximately symmetrical perceived intensity and discrimination for the right and left sides of the tongue. The perceptual asymmetry for most subjects was initially surprising, but there is evidence from previous studies that mechanical sensitivity in the oral cavity often differs on one side relative to the other (McCall and Cunningham, 1971; Lass et al., 1972). In fact, more recent studies indicate that most people have a chewing side preference during mastication and this preference is associated with better lingual somatosensory discrimination on the side ipsilateral to this preference (Minato et al., 2009). Interestingly multiple studies of randomly selected subjects indicate that right-side chewing preference may be more common than left-side chewing preference (Diernberger et al., 2008; Martinez-Gomis et al., 2009; Minato et al., 2009). Most of our subjects had better electrotactile perception on the right side of the tongue, and it is likely that this is due to increased over-all somatosensory perception in this region. Thus, many of our subjects likely had right-side chewing preference. In future studies, we plan to test this hypothesis by including subject interviews and mandibular kinesiograph measurements prior to electrotactile testing. If chewing preference is correlated with electrotactile perception, this would be another tool that could be used to design personalized mouthpieces for individualized lingual electrotactile sensory substitution devices. Together, our data and previous studies indicate that further study is needed to determine whether tongue sidedness to electrotactile stimulation is related to chewing side preference and/or structural differences in the oral cavity. In addition to chewing side preference, there is evidence that changes in oral structures such as tooth extraction and implants result in somatosensory cortical changes, which could affect perception (Henry et al., 2005; Avivi-Arber et al., 2011; Haggard and de Boer, 2014). It is feasible that differences in the palate or tooth structure could result in somatosensory differences between the right and left side of the tongue for some individuals.

Relative to electrotactile stimulation of other areas of the body, stimulation of the tongue is better able to convey information due to enhanced sensitivity and discrimination ability. Multiple factors contribute to these abilities including the protected environment of the mouth, the conductivity of saliva, and the properties of the epithelium, including a thinner cutaneous layer relative to skin (Bach-y-Rita et al., 1998; Bach-y-Rita and Kercel, 2003; Lozano et al., 2009). Mechanoreceptors innervating the tongue have small receptive fields and a corresponding large cortical area devoted processing information from these regions (Lass et al., 1972; Trulsson and Essick, 1997, 2010; Avivi-Arber et al., 2011). Careful microneurography studies conducted by Trulsson and Essick (1997, 2010) demonstrated that lingual mechanoreceptors can be classified into superficial and deep units, with superficial units further classified as rapidly or slowly adapting, and deep units consisting of only slowly adapting units (Trulsson and Essick, 1997). Local anesthesia disrupts mechanical two point discrimination ability, suggesting that superficial nerves contribute to this ability (Engelen et al., 2004). Based on the present studies and previous reports, the mechanoreceptors activated by electrotactile stimulation likely consist of the rapidly adapting, superficial units described by Trulsson and Essick (1997). The nerve fibers are easily depolarized by low voltage stimulation, have small receptive fields and most subjects describe the sensation of electrotactile tongue stimulation as a tingling sensation that disappears quickly.

The interesting finding that two electrodes are better discriminated if they are oriented horizontally (perpendicular the anterior-posterior axis if the tongue) is consistent with the neuroanatomy of the tongue. Mechanosensory information from the tongue is conveyed to the brain via the lingual nerve, which is part of the mandibular branch of the trigeminal nerve. Mu and Sanders (2010) examined adult human tongues with the goal to map out the hypoglossal nerve, a motor nerve supplying the tongue. Although the focus of their study was not the trigeminal nerve, the report contained valuable information about the course of this cranial nerve. In particular, they reported that individual bundles of fibers had receptive fields that always included the anterior tip of the tongue and individual fibers ran parallel to the longitudinal surface of the tongue before ending in the anterior tip. Based on this information, two electrodes oriented horizontally would be more likely to stimulate receptive fields of two different nerve fibers than two electrodes oriented vertically. In the latter case, the electrodes would be more likely to stimulate the same nerve fiber resulting in an inability to perceive the active electrodes as two separate stimuli. It should be noted that we have interpreted a higher mean perceived number value for certain conditions to mean that participants had better discrimination ability under those conditions. A source of error in this interpretation which was not predicted when this study was designed was that some participants reported a perceived number of 3 for certain stimuli. While this indicates that our interpretation of higher perceived number being equivalent to better discrimination ability contains errors, the effect of these anomalous points is minimal, as they account for less than 0.5% of participant responses.

A growing field of research considers the neuroanatomy and neurobiology of the tongue in the context of sensory substitution. Our current results add to work from other researchers who have labored to improve the perception of electrotactile stimuli on the tongue. In particular, this study compliments the work of Tyler et al. (2009) who studied the sensitivity of the tongue to electrotactile stimulation and that of Chekhchoukh and Glade (2012) who demonstrated methods to improve sustained electrotactile perception. Our research indicates that changes in discrimination ability on the surface of the tongue should be considered when designing electrode arrays to be used to communicate information to a person.

Novich and Eagleman (2015) have proposed a method for estimating the information throughput capability of a sensory substitution device using empirical data on an individual participant’s perception and discrimination ability of stimulus from the device. Moritz (2017) applied these ideas to the tongue and demonstrated that data from our studies can also be used to estimate the information throughput of the tongue using electrotactile stimulation. He suggested achievable throughput in terms of bits per second, or baud rates, for a number of study participants. Future studies could be performed which test these information throughput rates explicitly. It is likely that long-term exposure to relevant electrotactile tongue stimulation will result in an increased ability to detect and decipher signals sent via the trigeminal nerve of the tongue. The effects of training on the throughput ability of an individual’s tongue using electrotactile stimuli should be studied. Future studies using our methods of electrotactile stimulation should maintain standard time delays between active electrode pairs for consistency, as our current device allows for a range of time delays up to 35 ms between pulses on two simultaneously active electrodes. This could have an effect on the perception of the two stimuli.

## Author Contributions

JM, JDW and LMS-R designed the experiments, JM and LMS-R carried out the experiments and wrote the manuscript, PT analyzed the data and provided critical statistical analyses. JM and PT generated figures and tables. All authors critically reviewed the manuscript.

## Funding

These studies were funded in part by Colorado Office of Economic Development and International Trade (OEDIT) grants from the State of Colorado awarded to JDW (award no. HB 13-1193) and LMS-R (award no. ID #APP-065694), the OEDIT from the state of Colorado for LMS-R received a match from the City of Fort Collins and Sapien LLC. Additional funding was from internal awards from Colorado State University awarded to LMS-R (College Research Council award from the College of Veterinary Medicine and Biomedical Sciences and an award from the Provost office for providing experiential research for undergraduate students).

## Conflict of Interest Statement

JM and JDW have ownership in Sapien, LLC. JM, JDW and LMS-R are listed on the published patent entitled Tongue Stimulation for Communication of Information to a User, patent number WO 2015/167750 A2. PT reported no conflicts of interest.
